# Automated Detection of COVID-19 Using Deep Learning Approaches with Paper-Based ECG Reports

**DOI:** 10.1007/s00034-022-02035-1

**Published:** 2022-05-20

**Authors:** Mahmoud M. Bassiouni, Islam Hegazy, Nouhad Rizk, El-Sayed A. El-Dahshan, Abdelbadeeh M. Salem

**Affiliations:** 1 Egyptian E-Learning University (EELU), 33 El-messah Street, Eldokki, El-Giza, 11261 Egypt; 2 Faculty of Computer and Information Science, Ain Shams University, Abbassia, Cairo, 11566 Egypt; 3 Computer Science Department, Houston University, Houston, USA; 4 Department of Physics, Faculty of Science, Ain Shams University, Cairo, 11566 Egypt

**Keywords:** COVID-19 Diagnosis, Paper-based ECG image reports, Deep learning, Convolutional neural network (CNN), ECGConvnet

## Abstract

One of the pandemics that have caused many deaths is the Coronavirus disease 2019 (COVID-19). It first appeared in late 2019, and many deaths are increasing day by day until now. Therefore, the early diagnosis of COVID-19 has become a salient issue. Additionally, the current diagnosis methods have several demerits, and a new investigation is required to enhance the diagnosis performance. In this paper, a set of phases are performed, such as collecting data, filtering and augmenting images, extracting features, and classifying ECG images. The data were obtained from two publicly available ECG image datasets, and one of them contained COVID ECG reports. A set of preprocessing methods are applied to the ECG images, and data augmentation is performed to balance the ECG images based on the classes. A deep learning approach based on a convolutional neural network (CNN) is performed for feature extraction. Four different pre-trained models are applied, such as Vgg16, Vgg19, ResNet-101, and Xception. Moreover, an ensemble of Xception and the temporary convolutional network (TCN), which is named ECGConvnet, is proposed. Finally, the results obtained from the former models are fed to four main classifiers. These classifiers are softmax, random forest (RF), multilayer perception (MLP), and support vector machine (SVM). The former classifiers are used to evaluate the diagnosis ability of the proposed methods. The classification scenario is based on fivefold cross-validation. Seven experiments are presented to evaluate the performance of the ECGConvnet. Three of them are multi-class, and the remaining are binary class diagnosing. Six out of seven experiments diagnose COVID-19 patients. The aforementioned experimental results indicated that ECGConvnet has the highest performance over other pre-trained models, and the SVM classifier showed higher accuracy in comparison with the other classifiers. The resulting accuracies from ECGConvnet based on SVM are (99.74%, 98.6%, 99.1% on the multi-class diagnosis tasks) and (99.8% on one of the binary-class diagnoses, while the remaining achieved 100%). It is possible to develop an automatic diagnosis system for COVID based on deep learning using ECG data.

## Introduction

Coronavirus (COVID-19) first emerged in the Wuhan region of China in early December 2019. This virus causes respiratory infection and can be transmitted from one individual to another. The virus has spread all over the world since its appearance [23, 24]. It has been influencing life in various social, health, and economic aspects. In March 2020, the number of deaths worldwide because of this virus was huge, and by May 2021, there were more than 153 million people infected with COVID-19 all over the world. The number of people who recovered from this virus was 132 million, but more than 3.2 million died because of it [76]. Therefore, there is a need for an efficient and accurate diagnosis process to detect the infection with COVID-19. There are different types of protocols for the diagnosis announced by the World Health Organization (WHO). One of these protocols is the reverse transcriptase-polymerase chain reaction (rRT-PCR) [66]. Although PCR produces high accuracy, PCR tests require long waiting times normally from 4 to 6 h. Another protocol used for the diagnosis of COVID-19 is the radiography images. Based on the demerits of the PCR protocol, computed tomography (CT) and X-ray are applied for the early detection of COVID-19 [77]. The images obtained from the former protocol can provide important information in the diagnosis stage. Different studies use the radiographic image in the diagnosis of COVID-19 [2, 32, 51, 68]. Even though the studies obtained a high diagnosis rate, radiographic images have several drawbacks such as high cost, not being portable, requiring perfect skills in analysis and examination of the image, and high radiation exposure [12]. Based on applied protocols, new techniques are used as the COVID-19 continues in evolution. Not only does the impact of COVID-19 infect the respiratory system, but it also affects different organs in the human body. One of the main organs that are affected by this pandemic is the cardiovascular system. The heart rhythm is changed in the case of COVID-19 diagnosis [19]. One of the main signals that are influenced by changes due to COVID-19 is the electrocardiogram (ECG) [72]. The main findings in ECG signals of the COVID-19 patients are changes in ST [25], prolongation in QT [58], and shortening in PR intervals [9]. These findings lead to the ability of ECG signals to identify COVID-19 patients. Furthermore, the application of ECG brings a lot of advantages such as convenient cost, accessibility, monitoring of the changes in ECG in real time, and harmlessness. Automatic detection of COVID-19 using ECG signals will add a great value to PCR and radiography images.

In the fight against COVID-19, several methodologies and techniques are applied, such as the internet of things [63, 64] (IoT) and artificial intelligence [63, 64]. In addition to this, recent studies suggested the integration of AI into IoT to assist healthcare experts and patients [22, 29, 30]. One of the sub-branches of AI is deep learning (DL, and it is one of the recent methodologies that provide higher diagnostic performance compared to other techniques. DL can create a model without any manual feature extraction process compared to conventional machine learning (ML techniques. DL methods are fast and provide automatic detection of the disease, and the main superiority of DL methods is that they do not require expertise,therefore, they can help doctors and healthcare experts. They can learn from data and train on them efficiently to be able to provide an efficient diagnosis performance [52]. The former reasons led to the great popularity of DL in applications recently. Many DL approaches were applied for the automatic diagnosis of cardiac arrhythmia. Some studies used 1D ECG signals to train the deep learning models [11, 85]. Other studies converted 1D ECG signals to 2D representations using time–frequency and time-scale representations. Examples of the former representations are short-time Fourier transform (STFT) [27, 39], continuous wavelet transforms (CWT) [26], dual beat coupling matrices (DPCM) [86], and high-order spectral representation (HOSR) [1]. Moreover, paper-based ECG reports captured by doctors and healthcare professionals are largely used [7], but studies that provide an automatic diagnosis for cardiac problems are still significantly lacking. This study addresses four main contributions:

- Investigation of the ECG data in the paper-based form rather than using digital ECG signals.
- Proposal of automatic diagnosis systems for COVID-19 and other arrhythmias that occur in the ECG records.
- Introduction of a deep learning model known as ECGConvnet based on the hybridization between a pre-trained model and temporal convolutional network.
- Enhancement of the diagnostic accuracy compared with other proposed studies using ECG paper reports.

To achieve the former contributions, reasonable cost and automatic diagnosis models for COVID-19 and other disorders with high accuracy using DL are proposed in this manuscript. Firstly, the ECG data are collected from two recent publicly available online datasets. The data are preprocessed and augmented. Moreover, four pre-trained models based on DL are applied to the filtered and augmented data. Furthermore, a novel model named ECGConvnet based on the ensemble between the Xception model and TCN model is built. Then, a set of classifiers is used such as softmax, random forest (RF), multilayer perceptron (MLP), and support vector machine (SVM). Finally, a set of statistical performance measurements are calculated to evaluate the performance of the diagnosis models. The following paper is structured as follows: Sect. 2 presents a summary of the related work based on ECG data caused by COVID-19, while Sect. 3 explains the methodology based on data acquisition, filtration, augmentation, feature extraction using pre-trained models and the proposed model, and classification. In Sect. 4, the experimental and the classification results are manifested. Section 5 determines the discussion, Sect. 6 presents the applications, and Sect. 7 specifies the conclusions and future work.

## Related Works

Various studies have recently revealed that ECG can be applied for the diagnosis of COVID-19 due to the changes caused in signals. A study was proposed by Y. Wang et al. [73–75] on 319 COVID-19 patients with abnormal ECG heartbeats. It was manifested that there was a great change in the ST interval and the shape of the T wave. Another study was developed by Parvi et al. [53] on 75 patients with COVID-19 disease. It was found that 50.7% of the patients had their RR interval shortened and a large acceleration in the heart rate, but there was no change in the ECG signals in the remaining patients. Moreover, an experiment was done on people before and after COVID-19. In the experiment conducted, a great difference was illustrated in the heart rate and short PR interval in people with COVID-19. The study showed that the mortality rate is higher in patients that have their PR interval shortened. Moreover, Angeli et al. [3] performed experiments on 50 patients with COVID-19. They observed that there was an abnormal ST and left ventricular hypertrophy in 30% of the patients. Furthermore, different abnormalities were found in COVID-19 patients, such as tachy-brady syndrome, atrial fibrillation (AF), and acute pericarditis. It was also found that some COVID-19 patients had the right bundle branch block (RBBB) and myocardial (MI).

Li et al. [44] examined the ECG signals of 113 COVID-19 patients. Sixty-three of the patients survived, and the rest died. It was shown that ventricular arrhythmia existed more clearly in the patients who died than in the people who survived. Wide sinus tachycardia was observed in people who survived. Another study was conducted by Santoro et al. [59] on 110 patients and their ECG data. A clear prolongation in the QT interval in 14% of the patients was observed. In addition to this, Jain et al. [28] examined the ECG of COVID patients that used drugs as a treatment from COVID-19. There was a QT prolongation in their ECG signals. It was observed that the COVID-19 patients with abnormality had a higher rate of intubation than COVID-19 patients with normal heartbeats. McCullough et al. [47] applied an experiment on 756 COVID-19 patients. They detected a lot of abnormalities in these patients, such as intraventricular block, RBBB, and ST-elevation in some patients. It was also observed that all these patients had a high mortality rate. Another study proposed by Lam et al. [41] depended on the examination of 18 COVID-19 patients. It was found that several abnormalities existed in the COVID-19 patients, such as prolonged PR interval, the elevation of ST-segment, AF, RBBB, and depression PR interval. It was also realized that COVID-19 patients with abnormalities in ECG signal tend to have an increased severity, and they tend to stay longer in the hospital than COVID-19 patients without heart problems. To reveal the existence of abnormalities in different patients, a study was conducted by Bertini et al. [10] on 431 COVID-19 patients. It was observed that 93% of these patients had abnormalities. 22% of the patients had AF, 30% of the COVID-19 patients had a right ventricular pressure overload, whereas a prolongation in ST interval was detected in four patients. Finally, based on the related work, ECG signals can contribute to the early detection of the COVID-19. The main reason for this is the change observed in the ECG data without any cardiovascular history [49]. This poses two main challenges that are solved in the proposed study. The first challenge is that the changes that occur in COVID-19 patients can be similar to changes that occur to other patients with abnormal ECG signals and not diagnosed with COVID. This can make it difficult for any proposed system to differentiate between their diagnoses. The second challenge is the need for a system with high accuracy percentage to ensure the existence of COVID in the patient.

## Methodology

**Fig. 1:**
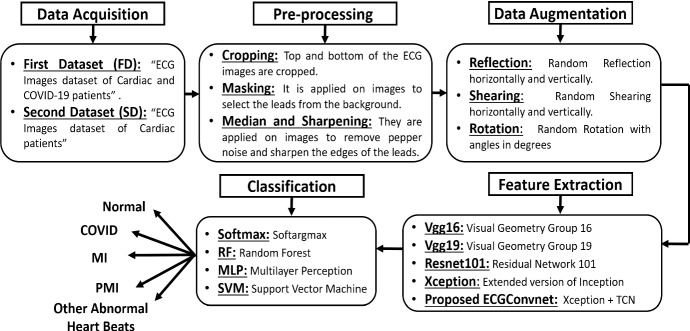
The proposed COVID-19 overall methodology using ECG images

The methodology consists of five main phases: data collection, filtering the ECG paper reports, augmenting the ECG reports, extracting most discernment features, and classifying the ECG reports. Figure 1 illustrates the whole methodology proposed for the diagnosis of ECG images. The data were collected from two online publicly available datasets.

The filtration phase of the ECG paper reports is based on cropping the images, masking, applying a medium filter, and sharpening the images. Then, the feature extraction phases are based on four pre-trained models which are Vgg16, Vgg19, Resnet101, and Xception. In addition to this, a novel deep learning model based on Xception and TCN is proposed, known as ECGConvnet. Finally, the classification is based on four popular classifiers known as softmax, RF, MLP, and SVM.

### Dataset

**Fig. 2:**
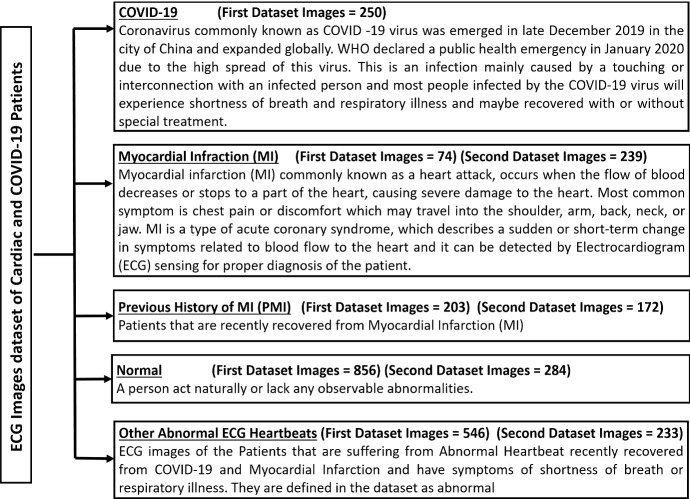
Full description of each ECG category collected in the dataset

The process of collecting and gathering data is a crucial step, and it must be specified accurately from the beginning. Two main datasets published recently are used in this study [37]. The first dataset (FD) is the “ECG images dataset of cardiac and COVID-19 patients” [35]. This dataset holds 1937 records collected using an ECG device known as “EDAN SERIES-3”. The data holds five categories: COVID-19, myocardial infarction (MI), previous history of MI (PMI), normal, and other abnormal ECG heartbeats. The ECG images hold 12 leads collected from patients in different cardiac institutes across Pakistan. The second dataset (SD) is the “ECG Images dataset of Cardiac patients [36]. This dataset holds four categories: MI, PMI, normal, and other abnormal ECG heartbeats. The images from both datasets are annotated by experts and healthcare professionals. Figure 2 represents the description of each ECG category in terms of symptoms, influence on the human body, and the number of ECG images in each category. The sampling rate and the number of leads of each category are defined by 500 Hz and 12 leads, respectively.

### Preprocessing

Preprocessing is a step that prepares the images in an enhanced form to be forward for feature extraction. Therefore, based on the ECG images obtained, it is found that four main stages are required for filtering. The four stages are cropping the ECG images, masking, median filter, and sharpening based on unsharp masking.

**Cropping**: All the ECG images are cropped from the top and bottom to remove unnecessary data that are not related to the ECG 12 leads. In the header and footer of the ECG image, some information is found about the patient number, gender, weight, room number, examination room, medication, diagnosis information, heart rate, date and time of the ECG report, and the common frequencies of the most important components in the ECG signal. All this information is cropped from the image to avoid any assistance that can provide knowledge about the type of diagnosis. The images are cropped by specifying a crop rectangle that holds only the 12 ECG leads of patients of different categories.

**Masking Filter:** This second stage is masking in which a mask is created to define the range of the black leads in the image. The mask is adjusted with a lower and upper bound. The lower and upper bounds are defined by 50 and 255, respectively. The mask creates an image with the leads colored in white and the background in black color. Finally, the “Not operation” is performed on the mask to obtain the leads in the black color and the background in white color. The resulting image is with leads in black color and white background.

**Median Filter:** It is the third stage in filtering; it is perceived to be one of the most effective filters in enhancing the images. Each ECG image resulting from the masking has “pepper noise” or some dark points; hence, a median filter is applied. It can be classified as a static nonlinear filter that can remove impulse noises easily. The operation of the medium filter starts by exchanging the value of the noise pixel with the median gray level located in the neighbor of this pixel [18]. A window size of 3 × 3 is applied on the ECG images to remove formerly mentioned noises. This operation makes the impulse noise position in the background disappear completely, but it can cause extra blurring to the edges. Finally, a sharpening filter is required to enhance the edge and make it as sharp as possible.

**Fig. 3:**
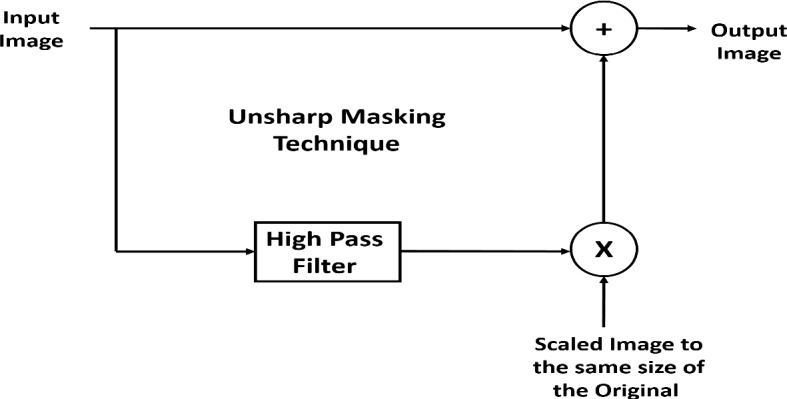
Unsharp Masking Technique

**Sharpening Filter:** In the proposed study, unsharp masking (UM) is applied extensively to sharpen the main edges and leads of the ECG image. The operation of unsharp masking UM is based on filtering the image using a high-pass filter, and the produced image is then scaled and summed to the original image to obtain the sharpened image [33]. Figure 3 shows the UM technique, which is applied to improve the medium contrast details. UM works using two main parameters: radius and amount. The radius is known as the standard deviation of the high-pass filter. The radius is used to control the size of the regions around the edge pixel that is going to be sharpened. If the value of radius is large, wider regions around the edge are sharpened. Otherwise, narrower regions around the edges are sharpened. The second parameter is the amount, and its typical values are from [0–5]. The higher the value of the amount, the higher the contrast of the sharpened pixels. Finally, the radius is adjusted to 5, while the value of the amount is 3.

**Fig. 4:**
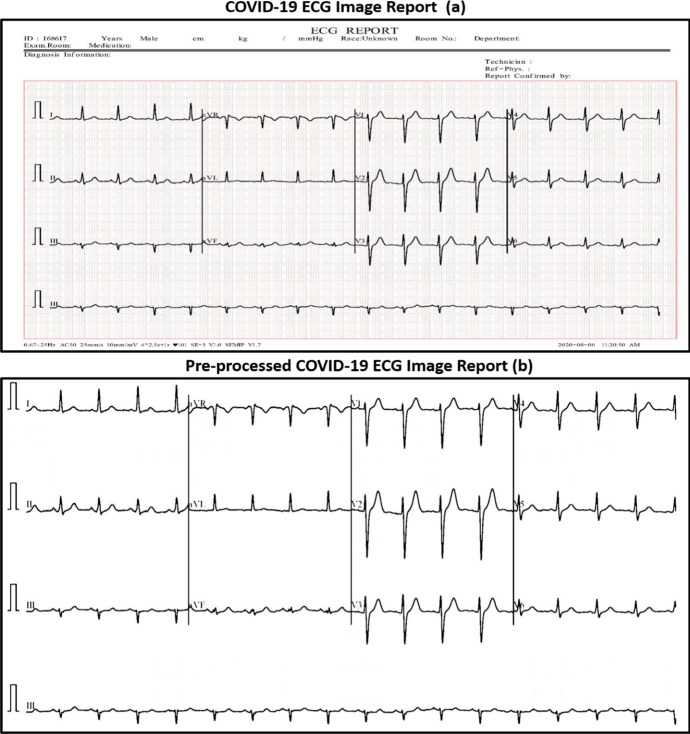
**a** COVID-19 ECG image report **b** Filtered COVID-19 ECG Image report

Figure 4 describes two main ECG image reports. Figure 4a represents a COVID-19 ECG image report from the dataset without any filtration process, whereas Fig. 4b shows the filtered COVID-19 ECG image report using the four proposed stages.

### Data augmentation

Data augmentation is a methodology that uses a suite of techniques to improve the quality and size of the training datasets. It is a very powerful technique in achieving a degradation in the validation error [17]. The data augmented manifest a set of different possible data that can reduce the distances between the training and the validation set as well as the testing set. The main role of data augmentation is the ability to prevent overfitting. It approaches overfitting from the root of the problem because it assumes that more information can be obtained from the original data using augmentations [61]. There are two main types of data augmentation: basic image manipulations and augmentation based on deep learning approaches.

The basic image manipulation approaches are based on kernel filters, geometric transformation, color space transformations, random erasing, and mixing images, whereas the deep learning approaches are based on adversarial training, neural style transfer, and generative adversarial networks (GANs) data augmentation. The data augmentation applied in this study is based on geometric transformations. The ECG image reports of the first dataset are augmented after being preprocessed based on a set of transformations, such as rotation, shearing, and reflection, while no augmentation is performed on the second dataset. As mentioned before, there are five ECG categories which are COVID-19, MI, PMI, normal, and abnormal. Table 1 shows the ECG categories, the augmentation operation performed on each category, and the number of images in each category after augmentation.

**Table 1:** Total number of ECG images after augmentation of the first dataset

Category no	Category name	Augmentation operation	No. of ECG images after augmentation
1	COVID-19	Random rotation [− 5 5]	750
		Shearing horizontal [− 0.05 0.05]	
2	MI	Random rotation [− 5 5]	747
		Random rotation [5 10]	
		Shearing horizontal [− 0.05 0.05]	
		Shearing vertical [− 0.05 0.05]	
		Shearing horizontal [0.1 0.2]	
		Shearing vertical [0.1 0.2]	
		Shearing horizontal [0.2 0.3]	
3	PMI	Random rotation [− 5 5]	812
		Shearing horizontal [− 0.05 0.05]	
		Shearing vertical [− 0.05 0.05]	
4	Normal	No augmentation	856
5	Abnormal	Random rotation [− 5 5]	750

### Feature Extraction Using Deep Learning Approaches

Feature extraction is performed to obtain features from the ECG images after the preprocessing and augmentation phases. A set of pre-trained models are applied to extract features and train on the ECG images. In addition, a novel model is proposed known as ECGConvnet. The five models are explained in detail in the following subsections.

#### Vgg16: Visual Geometry Group Architecture

The Vgg16 network architecture is proposed by Simonyan and Zisserman [62]. The models developed based on the visual geometry group are Vgg16 and Vgg19. Vgg16 consists of 41 layers. It is called Vgg16 because 16 layers from the 41 are with learnable weights,13 convolutional layers, and 3 fully connected layers. Vgg16 is a structured network that consists of five main blocks of convolutional layers and three fully connected layers. The first block consists of two convolutional layers [34]. Each convolutional layer is followed by a ReLU activation layer [56], and the second block is the same as the first block in structure. The third block consists of three convolutional layers. Each convolutional layer is followed by a ReLU activation layer, and the fourth and fifth blocks are the same as the third block in structure.

All the convolutional layers use 3 × 3 kernels with a padding of 1 and a stride of 1. At the end of each block, a max-pooling operation [73–75] is added. Each max-pooling layer has a kernel of 2 × 2 and a stride of 2, and no padding is performed. Then, three fully connected layers [8] are applied, two of them are with 4096 ReLU activation functions and the last one is with 1000 ReLU activation functions. Each fully connected layer is followed by a ReLU activation layer and a drop out layer [65], except the last fully connected layer which is followed by a softmax layer [31] and a classification layer [60].

#### Vgg19: Visual Geometry Group Architecture

Vgg19 is very similar to Vgg16, except that it has 47 layers and it is named Vgg19 because it has 19 layers with learnable weights [57]. The former layers are 16 convolutional layers and 3 fully connected layers. This model consists of five main blocks. The first block has two convolutional layers, and each layer is followed by a ReLU activation layer. The second block is similar to the first block in the structure of the layers. In the third block, four convolutional layers are defined, and each convolutional layer is followed by a ReLU activation layer. The fourth and the fifth blocks are similar to the third in the structure of the layer. The remaining layers of the model are the same as the Vgg16.

#### Resnet 101: Residual Neural Network

Resnet is considered one of the most interesting models in the computer vision and deep learning world [15]. Resnet stands for residual networks because it uses residual connections. In other words, the training of a few layers can be skipped based on residual connections. It is observed that it is easier to learn the residual of the output and input rather than the input only. Resnet 101 architecture consists of 33 blocks of layers, and all these blocks contain 104 convolutional layers and 104 batch normalization layers. The batch normalization layer applies normalization operation on the result obtained from the convolutional layers [20]. The addition layer is added at the end of each block to obtain the inputs of the next blocks [73–75].

The obtained residuals are the first operand to the addition layer, and the second operand is the inputs from the next blocks. The remaining 4 blocks use the outputs of the previous blocks and pass them to a convolutional layer with filter size 1 × 1 and stride 1. Then, the output is passed to the global normalization layer, and the resultant output is sent to the addition layer at the output of the final block. Finally, a global max-pooling layer is applied followed by a fully connected layer and a classification layer [67].

#### Xception

Xception is one of the powerful pre-trained models in deep learning [13]. The Xception model relies on grouped convolutional layers, sometimes known as depth-wise separable convolutional layers.

**Grouped Convolutional layer:** This layer separates the input channels into a set of groups, and sliding convolutional kernels are applied to these groups. In each group, the layer convolves the input entered bypassing the kernels on the input horizontally and vertically. After that, the dot product of the weight and the input is calculated and added to the bias. Independently, this layer coordinates the convolutions obtained from each group.

The effect of the group convolutional layer is that cross-channel and spatial correlations in the feature map of the CNN are decoupled completely. The Xception model consists of 36 convolutional layers that form the base of the feature extraction. The 36 convolutional layers are organized into 14 blocks having a linear residual connection around them, with the exclusion of the first and last blocks. The 14 blocks are divided into three main flows known as entry, middle, and exit flows.

**Entry Flow:** In this flow, the features are extracted using 8 convolutional layers followed by batch normalization layer and ReLU activation layer. The first two convolutional layers are standard convolutional layers, and the rest are group convolutional layers. In this flow, the filters in the group convolutional layers are twice the number of filters in the standard convolutional layers. The number of filters gradually increases by two. It starts with 64, then 128, and then finally 728 filters [55].

**Middle Flow:** This flow extracts more complex features through 24 grouped convolutional layers with 786 filters.

**Exit Flow:** The most detailed features are extracted in this flow using 4 group convolutional layers with 786, 1024, 1536, and 2048 filters, respectively. Finally, the last convolutional layer is applied with filter size 3 × 3, followed by a global average pooling layer to decrease the mapping size from 3 × 3 to 1 × 1, a fully connected layer, softmax layer, and classification layer.

#### The Proposed ECGConvnet Model

ECGConvnet is a proposed model based on the combination of the Xception model with the temporary convolutional network (TCN). The output of a fully connected layer in the exit flow of the Xception is an input to the proposed temporary convolutional model. The TCN model has shown its robustness over other deep learning models, such as CNN and RNN. TCN is recently used in several applications, such as probabilistic prediction, traffic forecasting, sound events detection, and many others. TCN was first proposed by Lea et al. [43], Lara-Benítez et al. [42] for the segmentation of actions from videos, and it showed high performance in segmentation, prediction, and classification. TCN works depending on two main steps. The first step is to compute the low-level features using CNN models, and these features hold spatial–temporal information. The second step is to forward the low-level features into a model that can be a CNN or RNN to capture the high-level temporal information. The proposed TCN takes the input data from the fully connected layer of the Xception model in the form of sequential features. The inputs of the TCN are mapped to a probability distribution. Then, the inputs are forwarded to four stacks of residual blocks. One residual block consists of 2 main dilated casual convolution layers, 2 weight normalization layers, 2 dropout layers, 1 ReLU activation layer, and 1 optional convolutional layer. Only the first residual block consists of three dilated casual convolution layers [21].

**Dilated Casual convolutional Layer:** TCN model can take a sequence of inputs with a specific length and produce the output with the same length as the input. It is called casual because the activations produced for a certain time step cannot rely on the activations from future time steps. The output of the fully connected layer of the Xception model is an input to the TCN defined by $Y = \left[ {y_{1} ,y_{2} , \ldots \ldots \ldots .,y_{i} } \right]$ and a filter $f:\left\{ {0, \ldots .., k - 1} \right\}$. The dilated casual convolutional operation on the $i$ point of $Y$ is defined using the following equation:

1 $$ C\left( {y_{i} } \right) = \mathop \sum \limits_{a = 0}^{k - 1} f\left( a \right) - y_{i - a.d} $$

where $d$ is the dilation factor, $k$ is the filter size, and $i - a.d$ represents the direction of the past. This means that the first layer maintains $Y$ as the input sequence, whereas in higher layers, $Y$ represents the output of the former layer. For each dilation convolutional layer, there is a dilation factor that increases exponentially by 2.

**Weight normalization Layer (WN):** This layer is applied for each dilated convolutional layer. The main aim of this layer is to separate the direction of the weight from the norm. Therefore, the weights have to be normalized by choosing a specific learning scaling parameter. The equation of the weight normalization operation is defined using the following equation:

2 $$  o_{j} = s_{j} \frac{{W_{j} * x}}{{\left| {\left| {W_{j} } \right|} \right|_{F} + }} + j $$

$$x$$ is the input of the WN layer,$$ o$$ is the output of the WN layer, $$s_{j}$$ is defined as the scale, $$j$$ determines the bias, ε is a constant that is used for numerical stability, $$W_{j}$$ and $$\left| {\left| {W_{j} } \right|} \right|_{F}$$ are the layer’s weight and the Frobenius norm of the weights for the output channel $$j$$, respectively, and $$*$$ is the convolution operator.

**Fig. 5:**
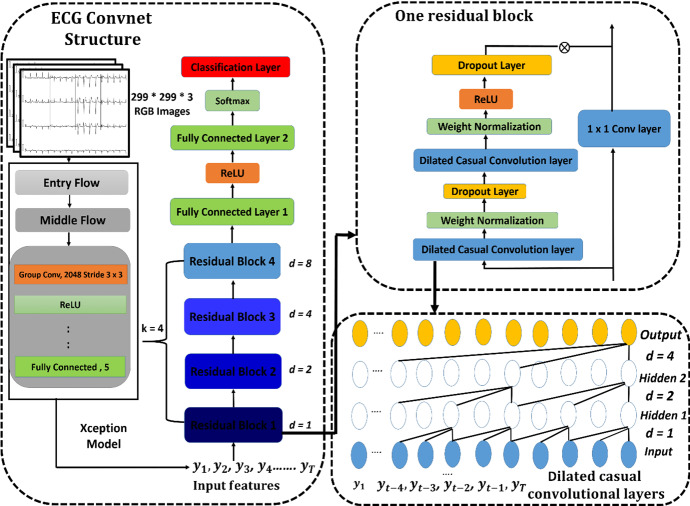
Proposed ECGConvnet

The input to the residual block is passed to 1-by-1 optional convolutional layer and then added with the output of the residual block. The main aim of this layer is to be applied on the input of the residual block when the number of channels of the output and input does not match. The same methodology is performed on the remaining residual blocks. Finally, after the four blocks are executed, the output of the fourth is passed to 2 fully connected layers, 1 ReLU activation layer, 1 softmax layer, and a classification layer. Figure 5 manifests the entire architecture of the ECGConvnet model.

Additionally, Table 2 describes the main parameters of the TCN and illustrates the number of blocks, dilation factors, and the number of input channels. It also determines the parameters of each dilated casual convolutional layer in terms of weight, bias, stride, and padding. Moreover, the figure shows the parameters of the last fully connected layer and the 1 × 1 convolutional layer. Finally, the length of our output will have the same length as the input.

**Table 2:** TCN blocks’ parameters

Proposed Model Layer Parameters	Assigned values experimentally		
Number of blocks	4		
Number of filters	175		
Filter size	3 × 3		
Dropout factor	0.07		
Number of input channels	5		
*ECGConvnet blocks*			
	Dilated Causal Conv 1	Dilated Causal Conv 2	Dilated Causal Conv 3
	Weights = 175 × 5 × 3	Weights = 175 × 5 × 3	Weights = 175 × 5 × 3
Block 1	Bias = 175 × 1	Bias = 175 × 1	Bias = 175 × 1
	Stride = 1	Stride = 1	Stride = 1
	Dilation factor = 1	Dilation factor = 1	Dilation factor = 1
	Padding = [2; 0]	Padding = [2; 0]	Padding = [2; 0]
	Dilated Causal Conv 1	Dilated Causal Conv 2	
	Weights = 175 × 175 × 3	Weights = 175 × 175 × 3	
Block2	Bias = 175 × 1	Bias = 175 × 1	
	Stride = 1	Stride = 1	
	Dilation factor = 2	Dilation factor = 2	
	Padding = [4; 0]	Padding = [4; 0]	
	Dilated Causal Conv 1	Dilated Causal Conv 2	
Block3	Weights = 175 × 175 × 3	Weights = 175 × 175 × 3	
	Bias = 175 × 1	Bias = 175 × 1	
	Stride = 1	Stride = 1	
	Dilation factor = 4	Dilation factor = 4	
	Padding = [8; 0]	Padding = [8; 0]	
Block4	Dilated Causal Conv 1	Dilated Causal Conv 2	
	Weights = 175 × 175 × 3	Weights = 175 × 175 × 3	
	Bias = 175 × 1	Bias = 175 × 1	
	Stride = 1	Stride = 1	
	Dilation factor = 8	Dilation factor = 8	
	Padding = [16; 0]	Padding = [16; 0]	
Optional 1 × 1 convolutional layer	Weights = 1 × 5 × 175		
	Bias = 175 × 1		
Fully connected	Weight = 5 * 175		
	Bias = 5 * 1		

### Classification

Classification is the last phase of the methodology, and it is the phase that tests the performance of the feature extraction models [6]. In this phase, four different classifiers are applied: softmax, RF, MLP, and SVM. Firstly, softmax is an extended version of the logistic regression [46]. It gives probabilities for each class label to determine the suitable class for each input test sample. Secondly, RF is a combination of tree classifiers [14]. Each tree classifier is developed based on a random vector that is sampled independently from the input data. Each tree produces a vote for the most famous class to verify the input. Thirdly, MLP is a machine learning classifier applied in many different applications and problems [16]. The main advantage of MLP is that it adds hidden layers to solve the problem of linear classification. MLP uses the backpropagation algorithm to transmit the error back to the hidden layers and update the weights and bias based on this error. Finally, SVM was proposed by Vapnik [71], and it has been conducted for classification, regression, and many other applications. The process of training SVM is a quadratic optimization problem. The main functionality of SVM is that it maps the input features into a high-dimensional feature field using a nonlinear function so that it can verify the test sample. The former classifiers are applied for the deep learning models to determine the highest performance classifier and the most robust model in feature extraction.

## Experimental Results

### Experimental Setup

This section illustrates the experiments applied on the five DL models using the ECG paper reports. These experiments are performed on a laptop with Intel Core i7- 8565 @1.80 GHz 1.99 GHz, 12 GB RAM. The graphic card specifications are NVIDIA GeForce GTX 310 M 2 GB. All the DL models were developed using MATLAB software. Additionally, the toolboxes and functions required to build the DL models were provided. Several experiments are performed for the diagnosis of the ECG heartbeats. These experiments are multi-class and binary-class classification problems. A multi-class experiment is performed for the diagnosis of five ECG heartbeats: COVID, MI, PMI, normal, and abnormal. In this experiment, all the ECG images were divided into three main sets: training, validation, and test. Fivefold cross-validation is performed using each of the five DL models. In each fold, the percentage of the training set is 60% of all data, while the validation and test set percentages are 20% and 20%, respectively. The total number of ECG report images is 3915, and they are divided into 2349, 783, and 783 for training, validation, and testing, respectively, in each fold.

### Hyperparameters and Classification Parameters Settings

The selection of the optimal hyperparameters is a complex task because each DL model has various parameters and it is difficult to select the optimal parameters of a model suitable for specific data. Therefore, a validation set was created based on 20% of the data to assist in selecting the optimal parameters for each DL model. If the average accuracy of the validation set after performing the five folds is satisfactory without causing overfitting on the training data, then the hyper-parameters of the model are preserved to the most suitable one selected for the model on the ECG data. Therefore, the model becomes ready to accept the test data. If the average accuracy of the validation set is not satisfactory, the hyperparameters are adjusted with different values till considerable accuracy is reached. The hyperparameters of the five models started with initial values, and then, they were updated by manual tuning until reaching the maximum validation accuracy. It is also important to consider that the training data are shuffled before each training epoch, and the validation data are shuffled before each network validation.

Table 3 shows the manual tuned hyperparameters of the five models in terms of network solver, gradient decay factor, learn rate drop factor, period, and schedule. Moreover, other parameters are adjusted, such as mini-batch size, iterations per epoch, initial learning rate, gradient threshold method and value, L2 regularization, and the maximum number of iterations.

**Table 3:** Hyperparameters of the five DL models

Hyperparameters training	Vgg16	Vgg19	Resnet101	Xception	Proposed ECG convent	
					Xception	TCN
Network solver optimizer	Sgdm	Sgdm	Sgdm	Rmsprop	Rmsprop	Adam
Mom/GDF/SGDF	Mom = 0.7	Mom = 0.4	Mom = 0.5	SGDF = 0.99	SGDF = 0.99	GDF = 0.990
						SGDF: 0.999
Max epochs	30	50	20	20	20	400
Iterations per epoch	391	391	391	391	391	1
Maximum iterations	11,730	19,550	7820	7820	7820	400
Mini batch size	8	8	8	8	8	1
Initial learning rate	$1{ } \times { }10^{ - 2}$	$1{ } \times 10^{ - 4}$	$1{ } \times 10^{ - 6}$	$1{ } \times 10^{ - 7}$	$1{ } \times 10^{ - 7}$	0.1
Learn rate drop factor	0.4	0.25	0.5	0.65	0.65	0.9
Learn rate drop period	2	4	2	2	2	5
Learn rate drop schedule	“Piecewise”	“Piecewise”	“Piecewise”	“Piecewise”	“Piecewise”	“Piecewise”
Gradient threshold method	“L2norm”	“L2norm”	“global L2-norm”	“global L2-norm”	“global L2-norm”	“global L2-norm”
Gradient threshold value	“Inf”	“Inf”	“Inf”	“Inf”	“Inf”	1
L2 Regularization	0.0005	0.0004	0.0002	0.0011	0.00011	0.0001
Verbose	1	1	1	1	1	0
Verbose frequency	100	100	100	100	100	50
Execution environment	‘gpu’	‘gpu’	‘gpu’	‘gpu’	‘gpu’	‘gpu’
Validation accuracy	96.4%	91.8%	97.5%	99.0%	99.6%	

The first parameter is the network solver optimizer, and this parameter is the solver for training the network. This parameter can hold one of three main functions: stochastic gradient descent momentum (Sgdm), root mean square propagation (Rmsprop), and adaptive moment estimation (Adam). In the Sgdm optimizer, the momentum parameter must be specified. In the Rmsprop optimizer, the decay rate of the square gradient descent factor (SGDF) must be determined, whereas, in the Adam optimizer, the decay rates of both SGDF and gradient descent factor (GDF) must be selected. The momentum is the contribution parameter that represents the update step of the previous iteration to the current iteration, while SGDF and GDF are the decay rates of the gradient and the squared gradient moving average, respectively. The values of SGDF and GDF are nonnegative scales less than 1, and they are denoted by $B_{1}$ and $B_{2}$, respectively.

The third to the fifth parameters are the number of epochs, iterations per epoch, and the total number of iterations. The sixth parameter is the mini-batch rate, and it represents the size of the batch for each training iteration. It is a subset of the training data that is used to estimate the gradient of the loss function and calculate the updates of the weights during training. The seventh parameter is the initial learning rate, and it is an important parameter that must be determined during the training. If the learning rate is high, then the training can result in a suboptimal solution or diverge. However, if the learning rate is low, training will take a long time. From the eighth to the tenth parameters, learning rate schedule, drop period, and drop factor can be found. In other words, the model has the option of keeping the initial learning rate the same during the entire training by allowing the learning rate schedule to be “none”, or the initial learning rate can be changed during iterations by allowing the learning rate schedule to be “piecewise”.

The learning rate drop period is the number of epochs for dropping the learning rate, while the learning rate drop factor is a multiplicative factor used to drop the learning rate. The gradient threshold method and the gradient threshold value are vital parameters. The gradient method is defined to clip the gradients that surpass the gradient threshold value. The gradient threshold method parameter can have one of two functions, which are “L2-norm” and “global L2-norm”. If the gradient method is “L2-norm” and the “L2-norm” of the gradient is larger than the gradient threshold, the gradient is scaled so that the L2-norm becomes equal to the gradient threshold. If the gradient method is “global L2-norm” and the “global L2-norm” is greater than the gradient threshold, all the gradients are scaled by a factor of gradient threshold/global 2-norm. Moreover, another parameter is the “L2 Regularization”, and it is known as the weight decay. It is a multiplier term for the weights to the loss function for reducing the overfitting. Finally, the verbose and the verbose frequency parameters are for displaying the training and validation results. This means that if the verbose value is one, the training and validation progress information will be displayed. Otherwise, it performs the training without any display of the progress, while the verbose frequency is the number of iterations between displaying to the command window. The validation accuracy calculated is based on the manually tuned parameters presented. Each fold is trained with 60% of the data and validated with 20% (not found in the train), and the remaining 20% (not found in the train or the validation) is left for the test. The same is performed for the remaining folds. Finally, the average validation accuracies of the five models of Vgg16, Vgg19, Resnet 101, Xception, and ECGConvnet after performing fivefold cross-validation on the former multi-class experiment are 96.4%, 91.8%, 97.5%, 99.0%, and 99.6%, respectively.

After adjusting the hyperparameters for training the DL models based on the validation set, the test set is input to the DL models for feature extraction and classification. The parameters of the classifiers should be selected carefully based on the highest accuracies obtained from them. Softmax has a single parameter that is the loss function. The next classifier is the random forest. It has some common parameters, such as the max depth of the tree, number of trees in the forest, number of features, and seed value that represents random numbers. If the maximum depth of the tree is 0, then the depth will be unrestricted. MLP has some main parameters, such as learning rate, number of epochs, and type of backpropagation. The type of backpropagation is momentum optimization back-propagation (MOBP), and a momentum parameter is adjusted to the weights during the iterations. Finally, the last classifier is SVM, and it has a set of main parameters, such as the batch size, calibration method, complexity parameter, kernel function, tolerance, and epsilon. The batch size is the size of the instances preferred to be processed when a batch prediction is applied. The calibration method is defined for a proper probability estimate. The predicted probabilities of SVM are coupled using a pairwise coupling method and then passed to the calibration. One of the main parameters of the SVM is the kernel function, and it is used to adjust the decision boundary. The tolerance and epsilon parameters are applied for round-off errors. The output of each of the five models is input to the four classifiers. Table 4 shows the classifiers and their corresponding parameters’ values used for the five models. It can be seen that some parameters have one value and other parameters have different values. The parameters with one value mean that if the value of the parameter is changed, it will decrease the performance of the folds of all models’ inaccuracy. Parameters that have different values illustrate that a model can have a value that maximizes its performance accuracy, and another model can have another value for the same parameter that maximizes the model’s performance.

**Table 4:** Classification parameters adjusted for the classifiers on the five models

Classifier	Parameters
Softmax	Loss function = “Cross Entropy Function”
Random forest (RF)	Max Depth = 0, Number of trees = [100,200,300,500]
	Seed = 1 Number of features = log2 (no. of predictors) + 1
Multilayer perception (MLP)	Learning rate = [0.05, 0.1, 0.2, 0.3]
	Backpropagation = MOBP
	Momentum = [0.1, 0.15, 0.2]
	Number of iterations = [500,1000,2000]
Support vector machine (SVM)	Batch Size = [50, 100, 200, 500]
	Calibration Method = “Logistic”
	Kernel function = “Polynomial function”
	Tolerance = 0.0001, Epsilon = [ 0, 1 x 10^-12^]
	Complexity parameter C = [1, 2, 4, 6]

### Classification Results

To prove that the proposed ECGConvnet shows the highest performance over other models, several statistical performance measurements [48] are conducted to evaluate its performance. These measurements start with accuracy (A), true positive (TP), false positive (FP), kappa statistic (K), true positive rate (TPR), precision (P), recall (R), F-measure (F1), Matthews’s correlation coefficient (MCC), receiver operation characteristics (ROC), and precision–recall curve (PRC) [40].

#### Classification results based on performance and statistical measurements

**Fig. 6:**
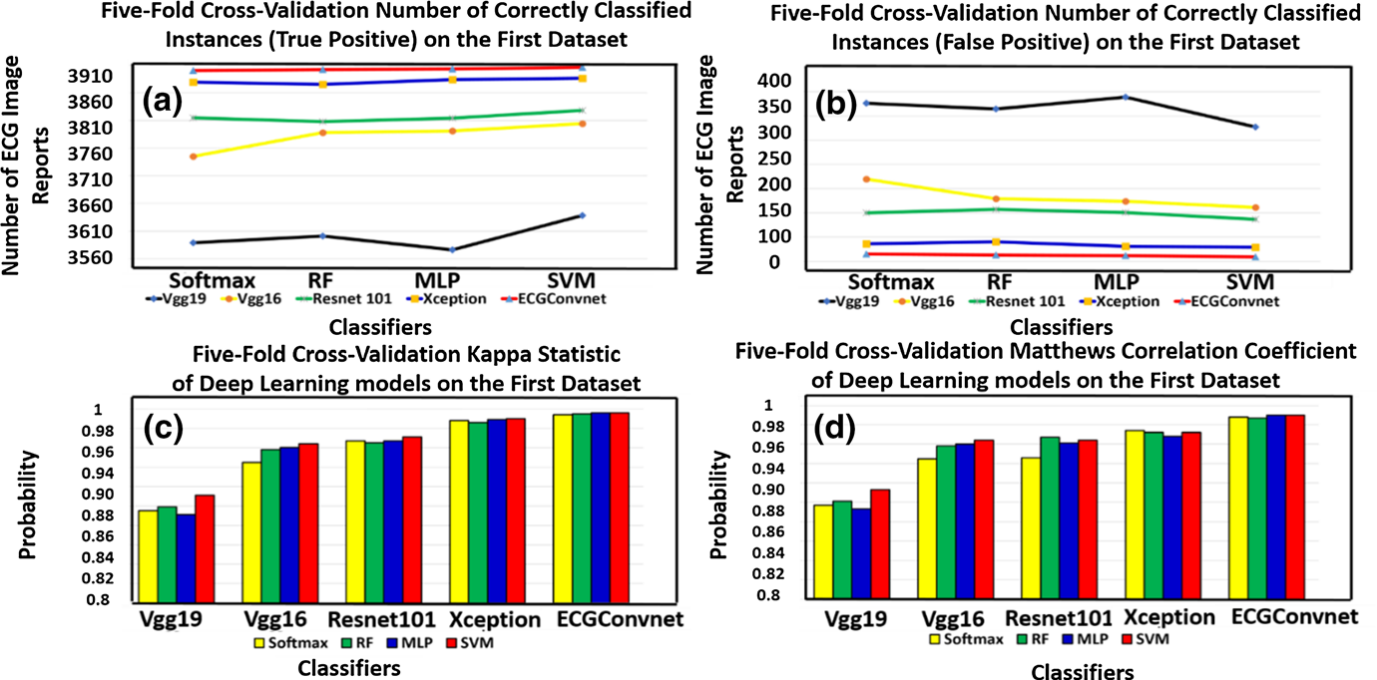
**a** and **b** The number of correctly and incorrectly classified instances after performing fivefold cross-validation, **c** and **d** represent the kappa statistic and Matthew correlation coefficient result over the five deep learning models

In this section, the results of the classifiers on the five models are illustrated in the former multi-class experiment. The test data are selected to be 20% of the overall data in each fold. The total number of ECG images is 3915, and each fold has about 783 ECG image test samples. Table 5 manifests the average statistical performance measurements after applying fivefold cross-validation on the deep learning models using four different classifiers. On the one hand, it can be verified from Table 5 that SVM and RF showed the highest performance on the Vgg19 model, while SVM and softmax manifested the highest performance on the Resnet101 model. On the other hand, it can be found that SVM and MLP showed the maximum performance on Vgg16, Xception, and ECGConvnet. Figure 6a and b illustrates a visualization for the number of correctly and incorrectly classified instances after performing a total of five folds. It can be seen that ECGConvnet has the highest performance in the number of ECG image reports classified correctly using the four classifiers, while Vgg19 showed the lowest performance. In terms of the results of the statistical measurements based on Fig. 6c and d, it can be manifested that ECGConvnet had the highest kappa statistic and Matthews’s correlation coefficient probability values.

**Table 5:** Classification results on the five DL models

Classifiers/models		Average fivefold cross-validation											
		A	TP	FP	K	TPR	FPR	P	R	F1	MCC	ROC	PRC
Softmax	Vgg16	91.69	3589	326	0.895	0.916	0.02	0.921	0.916	0.915	0.897	0.954	0.867
	Vgg19	95.66	3745	170	0.945	0.956	0.010	0.959	0.956	0.956	0.946	0.973	0.926
	Resnet101	97.44	3815	100	0.967	0.974	0.006	0.974	0.974	0.974	0.968	0.968	0.96
	Xception	99.07	3870	36	0.988	0.991	0.002	0.991	0.991	0.991	0.988	0.994	0.983
	ECGConvnet	99.64	3900	15	0.994	0.996	0.001	0.996	0.996	0.996	0.995	0.999	0.997
RF	Vgg16	91.97	3601	314	0.899	0.919	0.019	0.923	0.919	0.919	0.901	0.985	0.985
	Vgg19	96.72	3788	129	0.958	0.967	0.008	0.968	0.967	0.965	0.967	0.993	0.986
	Resnet101	97.26	3808	107	0.965	0.972	0.006	0.973	0.972	0.972	0.966	0.997	0.990
	Xception	98.9	3875	40	0.986	0.990	0.002	0.990	0.990	0.990	0.987	0.998	0.994
	ECGConvnet	99.66	3902	13	0.995	0.996	0.000	0.996	0.996	0.996	0.996	0.999	0.996
MLP	Vgg16	91.36	3576	339	0.891	0.913	0.020	0.917	0.913	0.913	0.893	0.981	0.948
	Vgg19	96.85	3791	124	0.960	0.968	0.007	0.969	0.968	0.966	0.961	0.996	0.990
	Resnet101	97.41	3814	101	0.967	0.974	0.006	0.974	0.974	0.974	0.968	0.996	0.991
	Xception	99.20	3884	31	0.989	0.992	0.001	0.992	0.992	0.992	0.990	0.997	0.989
	ECGConvnet	99.69	3903	12	0.996	0.997	0.008	0.997	0.997	0.997	0.996	0.999	0.999
SVM	Vgg16	92.94	3638	277	0.911	0.929	0.017	0.933	0.929	0.928	0.913	0.966	0.891
	Vgg19	97.16	3804	111	0.964	0.971	0.007	0.972	0.971	0.971	0.964	0.989	0.955
	Resnet101	97.79	3828	87	0.971	0.977	0.005	0.978	0.977	0.977	0.972	0.992	0.965
	Xception	99.25	3886	29	0.990	0.992	0.002	0.992	0.992	0.992	0.990	0.997	0.991
	ECGConvnet	99.74	3906	9	0.996	0.997	0.000	0.997	0.997	0.997	0.997	0.998	0.996

**Fig. 7:**
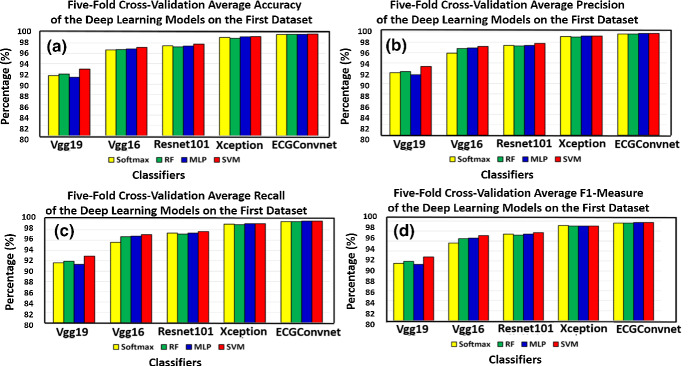
**a** and **b** The accuracy and precision, **c** and **d** represent the recall and F1-measure after performing fivefold cross-validation

Figure 7a and b represents accuracy and precision, whereas (c) and (d) represents the recall and F1-measure performance of the fivefold cross-validation using the deep learning models based on the four classifiers. It can be seen that the SVM classifier has the highest performance over all other classifier models, while ECGConvnet achieves the maximum improvement over other deep learning models in terms of the former measurements.

#### Classification Results Based on ROC and Confusion Matrices

**Fig. 8:**
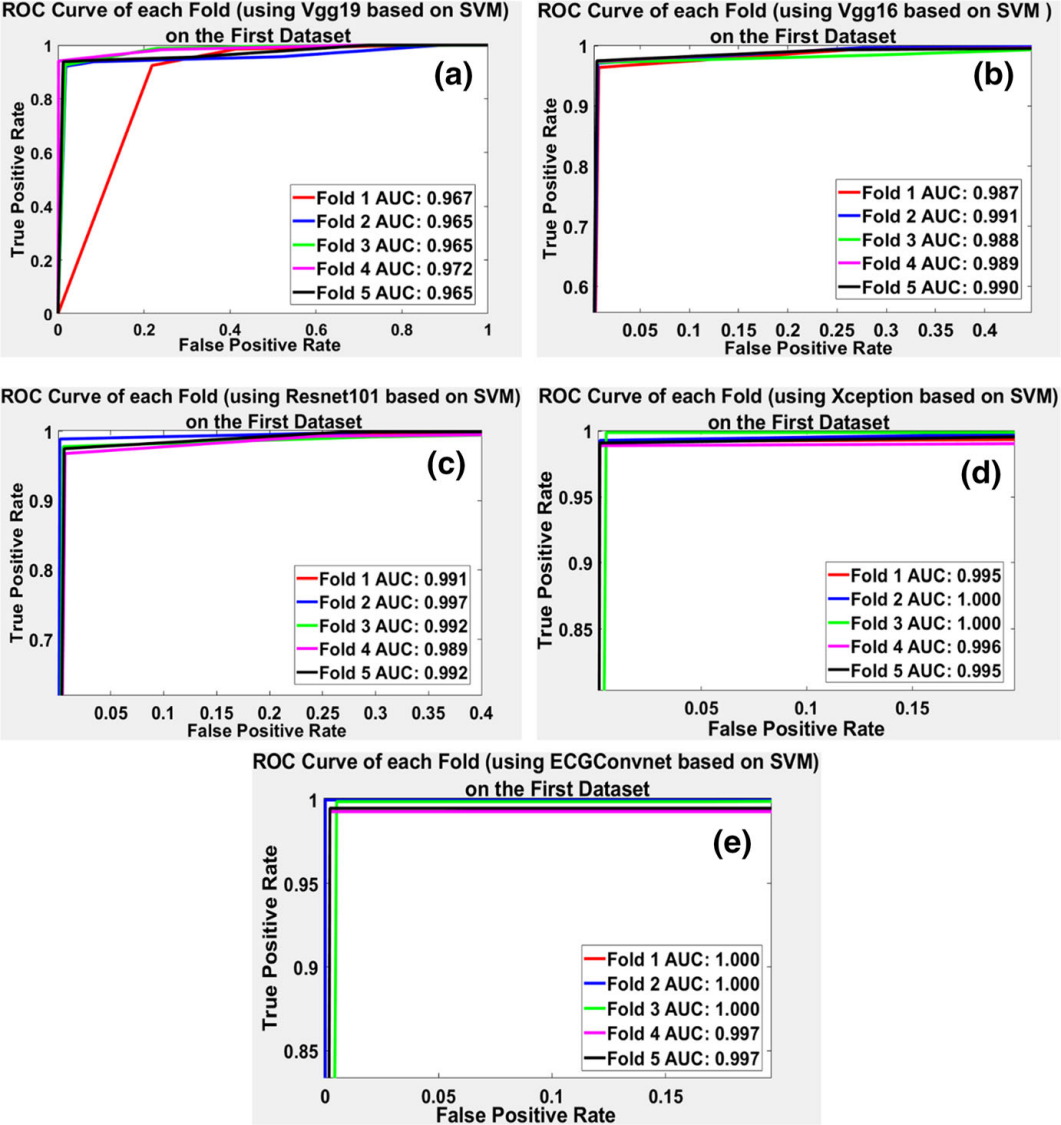
**a**–**e** The ROC curves of each fold based on the proposed deep learning models using SVM classifiers

The receiver operating characteristic (ROC) shows the performance of a classification model at all classification thresholds. To show the results of each fold of the fivefolds, ROC was drawn to investigate the performance of the deep learning model fold by fold. As mentioned before, the SVM showed the highest classification performance. Therefore, the ROC is illustrated based on the deep learning models using the SVM classifier. Figure 8a–e displays the ROC on each fold represented by the curves and the legends of the area under the curve (AUC) at each fold. It can be manifested that the ROC curves reached their maximum using ECGConvnet and Xception models in Fig. 8d and e.

**Fig. 9:**
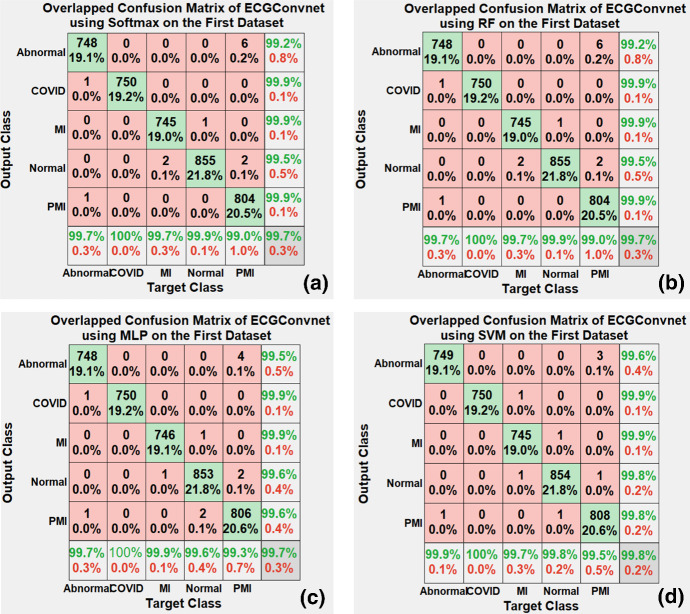
**a**–**d** The overlapped confusion matrix of fivefold results using the four classifiers on ECGConvnet

The worst performance was the use of Vgg19, while Vgg16 and Resnet101 showed an average performance on each of the five folds. This section also illustrates the confusion matrices obtained from the four classifiers in the deep learning models. The main aim of the confusion matrix is to describe the performance of the classifiers on the test data. It shows an overall accuracy of the performance of the classifier. The overlapped confusion matrices in Fig. 9 represent the performance of the proposed ECGConvnet model on the four classifiers after performing fivefold cross-validation. Let us consider the confusion matrix (c) in Fig. 9.

The confusion matrix has an x-axis that represents the target classes, and the y-axis represents the predicated classes resulting from the classifier. The green areas and the red areas describe correctly and the incorrectly classified images, respectively. The first row has 6 main cells. The first cell has an integer and a percentage. The integer represents the number of correct instances, which are 748 from the abnormal class, while the percentage is calculated based on dividing 748 over the total number of images, which is 3915. The cell before the last in the first row represents the number of incorrectly classified instances, which are 4 from the PMI class. The same concept is applied for rows from 2 to 5. The last row in the confusion matrix has cells; each cell has two percentages.

The first cell has a green percentage that represents precision, and the red percentage is the false discovery rate for the abnormal class. The same is done with the remaining cells for their corresponding classes. The last column in the confusion matrix also has cells; each cell has two percentages. The first cell has a green percentage that represents the sensitivity, and the red percentage is the false-negative rate for the abnormal class. The same is done for the remaining cells for their corresponding classes. Finally, the last cell in the down corner of the confusion matrix has two percentages colored in green and red. The green represents the overall percentage of the correctly classified ECG images, and the red is the overall percentage of the incorrectly classified ECG images.

#### More Experiments Based on ECGConvnet Model

Based on the previous subsections, it can be experimentally visualized that ECGConvnet had the highest and most accurate features compared to other deep learning models. Moreover, the SVM classifier showed the highest performance on the first dataset compared to other classifiers. Therefore, to prove the robustness of the ECGConvnet with SVM, six experiments are applied for the diagnosis of the ECG heartbeats. Fivefold cross-validation is applied to the following experiments. The first experiment is a multi-class classification problem, and it is based on the diagnosis of four different classes, which are MI, PMI, normal, and abnormal on the whole second dataset. This dataset holds 928 images divided into 594 images for training, 148 for validation, and 186 for testing in each fold. The second experiment is the diagnosis of three different classes: normal, COVID, and other abnormalities. The total number of images in this experiment is 750, and they are obtained from the first dataset. The images are distributed as follows: 250 normal, 250 COVID, and 250 from 125 abnormal and 125 MI classes. Fivefold cross-validation is performed on the data of this experiment. Each fold has 450 images for training, 150 images for validation, and the same for testing.

The third experiment is the diagnosis of COVID vs No Findings (normal) class, while the fourth experiment is the diagnosis of the positive (COVID) vs negative (other classes such as normal, abnormal, and MI). In the third experiment, 250 COVID and 250 normal ECG image reports are selected, while in the fourth experiment, 250 COVID, 83 normal, 83 abnormal, and 84 MI are chosen. In the second, third, and fourth experiments, images are selected from the first dataset without augmentation. The fifth and sixth experiments are similar to the third and fourth, except that the No Findings and negative classes are selected from the second dataset. From the third to the sixth experiments, the data are also divided into 60% training, 20% validation, and 20% testing. Each fold in the last four experiments consists of 400 training ECG images reports, 100 validation, and 100 testing. Each experiment is trained with the same parameters of ECGConvnet defined in Table 3. The average validation accuracy obtained from the first and the second experiments is 98.642% and 99.192%, respectively, while the remaining experiments obtained a validation accuracy of 100% using ECGConvnet. Table 6 represents the results of the former experiments. The table shows the details of each fold in the experiments. Various statistical performance measurements are calculated for each fold in each experiment. It can be seen that three experiments showed a perfect accuracy performance for the statistical measurements. The three experiments are COVID vs (No Findings of the first dataset), COVID vs (No Findings of the second dataset), and positive vs (negative of the second dataset).

**Table 6:** The results of six experiments fold by a fold in terms of statistical performance measurements using ECGConvnet

Experiments/folds		Fivefold cross-validation based on ECGConvnet with SVM classifier											
		A	TP	FP	K	TPR	FPR	P	R	F1	MCC	ROC	PRC
MI vs PMI vs normal vs abnormal	Fold1	98.92	184	2	0.985	0.989	0.004	0.989	0.989	0.989	0.986	0.994	0.985
	Fold2	100	186	0	1.000	1.000	1.000	1.000	1.000	1.000	1.000	1.000	1.000
	Fold3	97.31	181	5	0.963	0.973	0.008	0.974	0.973	0.973	0.965	0.991	0.960
	Fold4	100	185	0	1.000	1.000	1.000	1.000	1.000	1.000	1.000	1.000	1.000
	Fold5	96.75	179	6	0.956	0.968	0.012	0.968	0.968	0.967	0.957	0.999	0.997
Normal vs COVID vs other abnormalities	Fold1	100	150	0	1.000	1.000	1.000	1.000	1.000	1.000	1.000	1.000	1.000
	Fold2	99.33	149	1	0.990	0.993	0.003	0.993	0.993	0.993	0.990	1.000	1.000
	Fold3	100	150	0	1.000	1.000	1.000	1.000	1.000	1.000	1.000	1.000	1.000
	Fold4	97.33	146	4	0.960	0.973	0.013	0.975	0.973	0.973	0.961	0.989	0.984
	Fold5	98.66	148	2	0.980	0.987	0.007	0.987	0.987	0.987	0.980	0.999	0.999
COVID vs (no-findings of first dataset)	Fold1	100	100	0	1.000	1.000	1.000	1.000	1.000	1.000	1.000	1.000	1.000
	Fold2	100	100	0	1.000	1.000	1.000	1.000	1.000	1.000	1.000	1.000	1.000
	Fold3	100	100	0	1.000	1.000	1.000	1.000	1.000	1.000	1.000	1.000	1.000
	Fold4	100	100	0	1.000	1.000	1.000	1.000	1.000	1.000	1.000	1.000	1.000
	Fold5	100	100	0	1.000	1.000	1.000	1.000	1.000	1.000	1.000	1.000	1.000
Positive vs (negative of first dataset)	Fold1	100	100	0	1.000	1.000	1.000	1.000	1.000	1.000	1.000	1.000	1.000
	Fold2	100	100	0	1.000	1.000	1.000	1.000	1.000	1.000	1.000	1.000	1.000
	Fold3	100	100	0	1.000	1.000	1.000	1.000	1.000	1.000	1.000	1.000	1.000
	Fold4	99	99	1	0.98	0.990	0.010	0.990	0.990	0.990	0.980	0.990	0.985
	Fold5	100	100	0	1.000	1.000	1.000	1.000	1.000	1.000	1.000	1.000	1.000
COVID vs (no-findings of second dataset)	Fold1	100	100	0	1.000	1.000	1.000	1.000	1.000	1.000	1.000	1.000	1.000
	Fold2	100	100	0	1.000	1.000	1.000	1.000	1.000	1.000	1.000	1.000	1.000
	Fold3	100	100	0	1.000	1.000	1.000	1.000	1.000	1.000	1.000	1.000	1.000
	Fold4	100	100	0	1.000	1.000	1.000	1.000	1.000	1.000	1.000	1.000	1.000
	Fold5	100	100	0	1.000	1.000	1.000	1.000	1.000	1.000	1.000	1.000	1.000
Positive vs (negative of second dataset)	Fold1	100	100	0	1.000	1.000	1.000	1.000	1.000	1.000	1.000	1.000	1.000
	Fold2	100	100	0	1.000	1.000	1.000	1.000	1.000	1.000	1.000	1.000	1.000
	Fold3	100	100	0	1.000	1.000	1.000	1.000	1.000	1.000	1.000	1.000	1.000
	Fold4	100	100	0	1.000	1.000	1.000	1.000	1.000	1.000	1.000	1.000	1.000
	Fold5	100	100	0	1.000	1.000	1.000	1.000	1.000	1.000	1.000	1.000	1.000

It can also be observed that in the multi-class diagnosis based on the second dataset, the second and fourth folds showed the highest performance over others, while in the multi-class diagnosis based on three classes, the first and the third folds showed the highest performance measurements over other folds.

Finally, in the positive vs (negative of the first dataset) experiment, all folds showed the highest performance, except the fourth fold. It is also important to mention that the pre-trained models used in this study are applied to the first experiment based on the whole second dataset using the four classifiers. The SVM classifier had the highest accuracy performance over other classifiers. The accuracies obtained on the Vgg16, Vgg19, Resnet101, Xception using SVM classifier are 96.120%, 95.903%, 96.874%, and 97.735%, respectively.

**Fig. 10:**
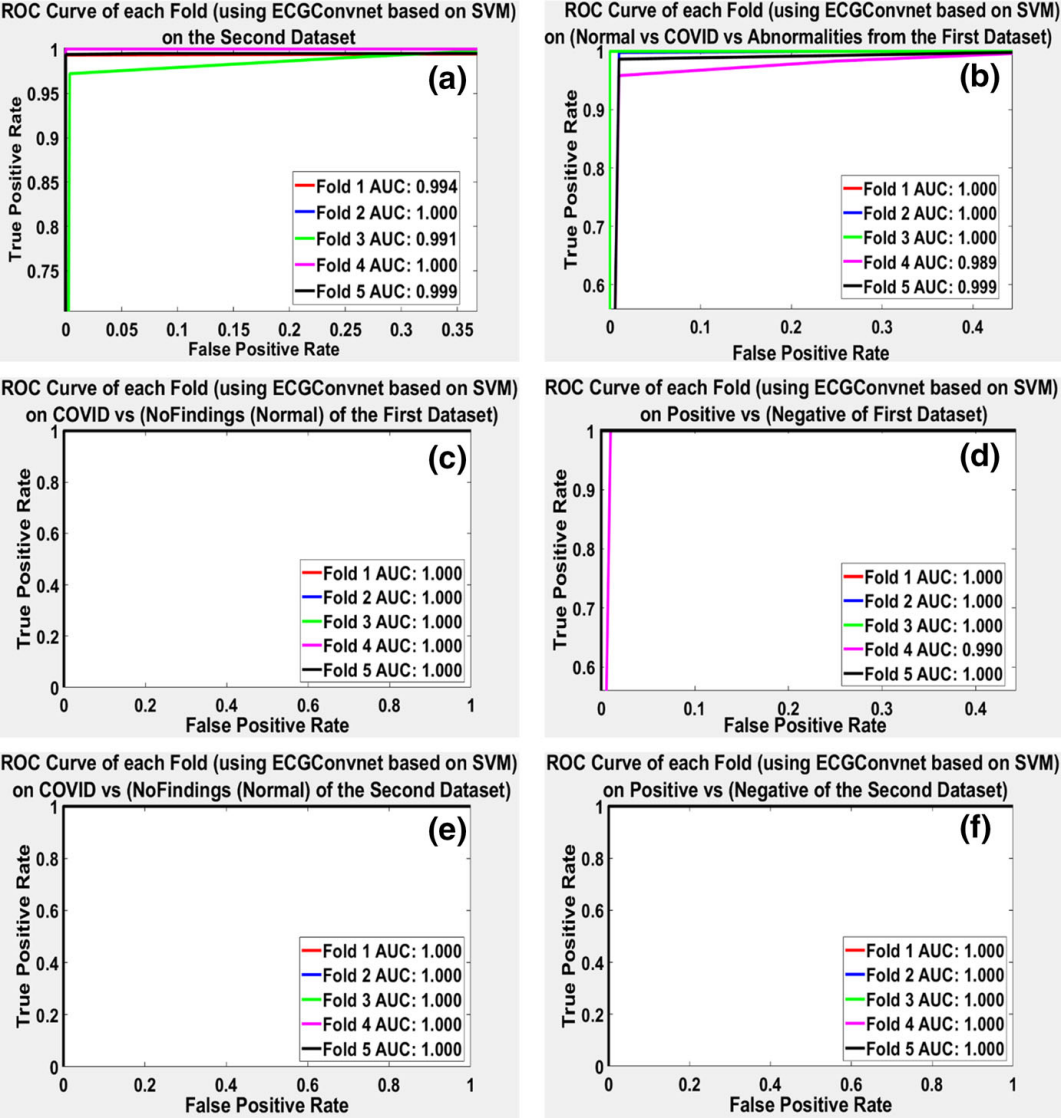
**a**–**f** The ROC of the five folds resulting from six experiments using ECGConvnet

Figure 10a–f shows the ROC of each fold of the six experiments. It can be illustrated that most of the folds in the six experiments showed a perfect AUC value. Some experiments showed a reduced value in the AUC. This is observed in the multi-class based on the second dataset, the multi-class based on three classes, and the positive vs (negative of the first dataset) experiments. It can be seen that the fourth fold in multi-class based on three classes experiment showed the lowest AUC. Figure 10a shows that the first, third, and fifth folds had the lowest AUC of values 0.994, 0.991, and 0.999, respectively, while Fig. 10b shows that the fourth and the fifth folds have the lowest AUC with a value equal to 0.989 and 0.999, respectively. Finally, in Fig. 10d, the fourth fold showed the lowest AUC with a value equal to 0.990.

**Fig. 11:**
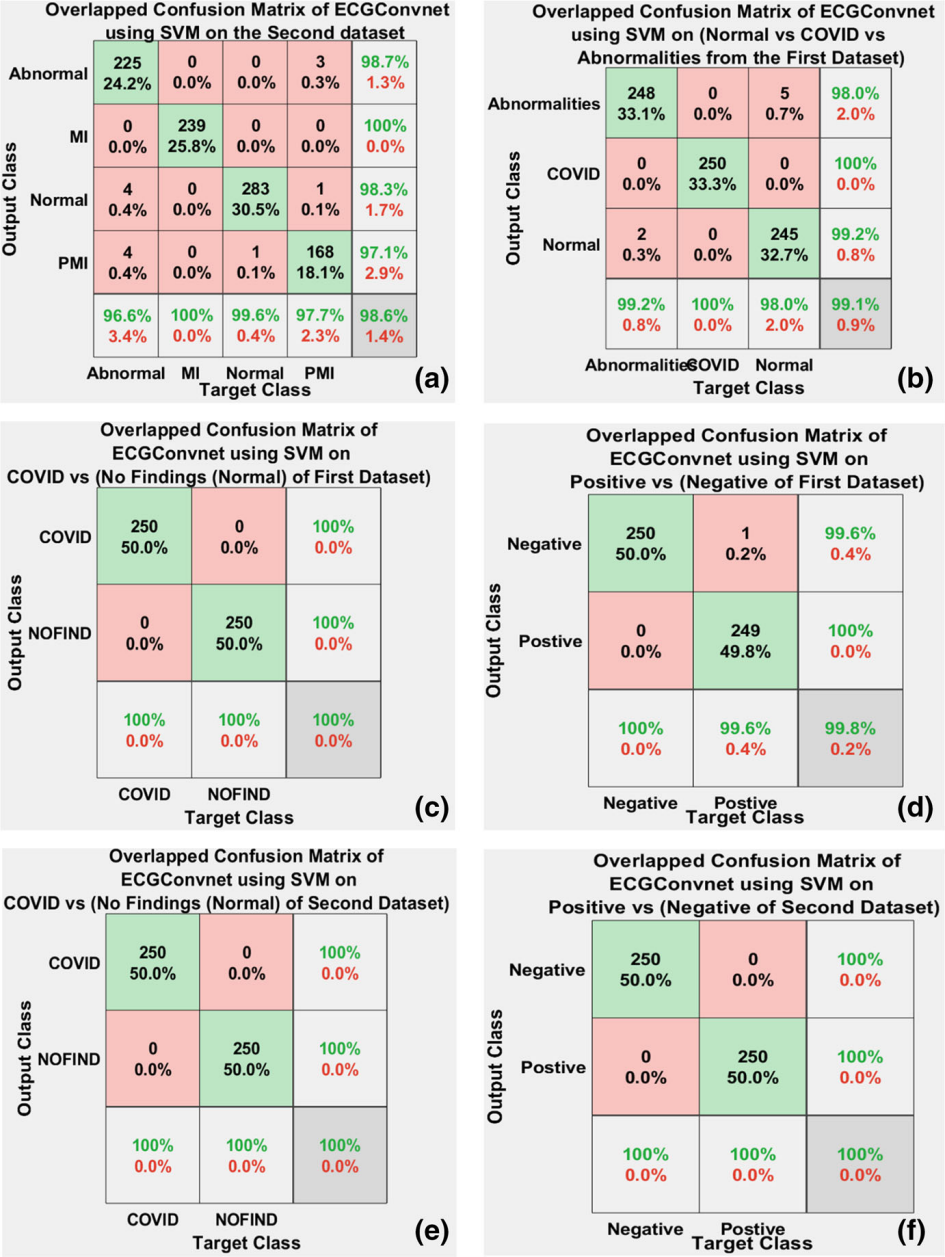
**a**–**f** The overlapped confusion matrices resulting from six experiments based on fivefold cross-validation using ECGConvnet

Figure 11a–f manifests the overlapped confusion matrices resulting from the six experiments based on fivefold cross-validation. COVID vs (No Findings of the first dataset), COVID vs (No Findings of the second dataset), and positive vs (negative of the second dataset) showed an accuracy of 100%. It is shown in Fig. 11a that 13 ECG paper reports are misclassified leading to an accuracy of 98.6%. In Fig. 11b, it can be recognized that 7 ECG paper reports are wrongly classified reaching an accuracy of 99.05%. Finally, in Fig. 11d, it is also discovered that in the positive vs (negative of the first dataset) experiment, one positive case (COVID) is misclassified as negative leading to an accuracy of 99.8%.

#### Data and Features Visualization based on ECGConvnet Model

It is salient to visualize the ECG paper-based reports data before and after extracting features from them. This visualization will provide information about the efficiency of the ECGConvnet model in the feature extraction, and whether the classifiers can diagnose or provide accurate detection of different diseases. Therefore, an algorithm known as t-distribution stochastic neighbor embedding (t-SNE) (Van der Maaten [70] is applied to achieve the requested visualization. t-SNE is an algorithm for dimensionality reduction, and it is suitable for visualizing high-dimensional data. This algorithm creates a set of low-dimensional points from the high-dimensional data. The low-dimensional points are visualized as natural clusters representing high-dimensional data.

To prove the efficiency of the proposed model, the t-SNE is drawn for the worst folds that showed the lowest accuracy performance in the two multi-class experiments with the largest number of images conducted in the study. On the one hand, it can be seen from the ROC in Fig. 8e that fold 4 showed the lowest performance when the ECGConvnet was applied for the diagnosis of COVID, MI, PMI, normal, and abnormal from the first dataset. On the other hand, it can be seen from Fig. 10a that fold 3 showed the lowest performance when ECGConvnet was applied for the diagnosis of MI, normal, PMI, and abnormal from the second dataset.

**Fig. 12:**
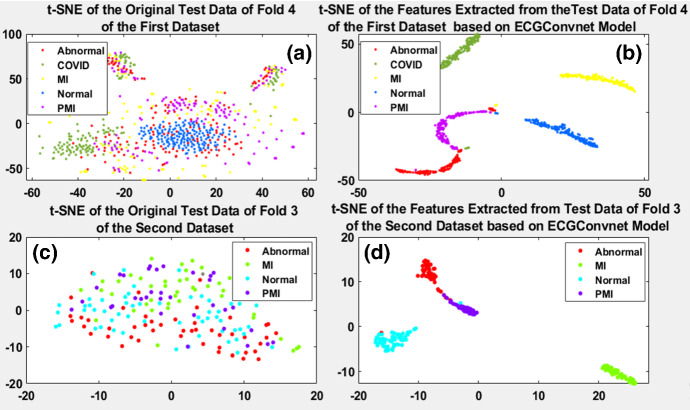
**a** and **b** Low-dimensional points representing fold 4 and fold 3 test ECG paper-based reports in their original form gathered from the first, and the second dataset **c** and **d** shows low-dimensional points representing fold 4 and fold 3 test ECG paper-based reports after applying ECGConvnet model on them

Therefore, t-SNE is drawn before and after applying ECGConvnet on them. Figure 12a shows fold 4 original test data of ECG reports of the first dataset before the application of any processing on it, while Fig. 12b shows the features resulting from the ECGConvnet model on fold 4 test data. The same is done and shown in Fig. 12c and d on fold 3 test data of the second dataset. It can be manifested in Fig. 12b and d that the features obtained from ECGConvnet can be separated and classified easily using various classifiers. This proves the robustness of the ECGConvnet.

## Discussion

The proposed work demonstrates the effectiveness of ECG image reports in the diagnosis of different heart diseases, especially the COVID-19 virus. The proposed methodology consists of five main phases: data acquisition, preprocessing, augmentation, feature extraction using deep learning models, and classification. The first phase is obtaining the ECG image reports data. The data are collected from two datasets known as the “ECG Images dataset of cardiac and COVID-19 patients” and “ECG Images dataset of Cardiac patients.” The first dataset consists of five ECG classes, which are COVID, MI, PMI, normal, and abnormal, while the second dataset consists of four ECG classes which are MI, PMI, normal, and abnormal. There are three reasons for the selection of the former datasets. The first reason is that these datasets are recently published and available online in 2021. The second reason is that one of them holds ECG COVID patients and holds ECG 12-lead image reports rather than samples of ECG signals. The last reason is that they can show how the proposed deep models perform on the COVID diagnosis using ECG reports. The second phase is the filtering or the preprocessing phase. In this phase, four filtering stages are performed: cropping, masking, medium filter, and unsharp masking filters. There are several reasons for the usage of the former preprocessing stages. The first reason is to remove the header and the footer of the ECG image and keep the leads only without any information that can assist in the diagnosis process. The second reason is to remove the pepper noise and the colored squares in the ECG images. The final reason is to sharpen the ECG leads and their neighbors so that the images can be enhanced for further processing. The next phase is the data augmentation phase, and this phase is performed for two main reasons. The first reason is to avoid overfitting data. The second reason is to increase the number of images in each ECG class to balance each class in the number of ECG images. Therefore, in the classification stage, the division of the training, validation, and testing will be nearly equivalent in each class.

The fourth phase is the feature extraction phase. In this phase, five deep learning models are presented. The first four models are transfer learning models, such as Vgg16, Vgg19, Resnet101, and Xception. The fifth model is a proposed model defined by ECGConvnet. The former transfer learning models are selected because they have proved their classification performance as they achieved top-1 and top-5 accuracy in classification. Additionally, the proposed model ECGConvnet is built based on Xception and TCN. The Xception was chosen to build the ECGConvnet structure because it has shown the highest performance over other transfer learning used on both datasets. The fifth phase and last phase is classification. In this phase, softmax, RF, MLP, and SVM classifiers are selected. It can be seen that SVM had the highest accuracy in all models. Seven experiments are performed to verify the efficiency of the deep learning models, especially the ECGConvnet. Some of them are multi-class tasks and the remaining are binary-class. The aim is to concentrate on the ability to diagnose COVID concerning other ECG diseases. It can also be deduced from the experiments that ECGConvnet showed the highest performance. Moreover, several qualitative and quantitative statistical measurements are applied to provide accurate performance results.

The complexity of any deep learning model depends on various factors, such as learning time, amount of data, layers of the model, as well as number and size of filters in each layer. The proposed model complexity is evaluated based on estimating the learning time, extracting features from the fully connected layers, and classifying the test data in each fold. The training time of the proposed model on the three multi-class experiments is 3.75, 1.65, and 1.2 h, respectively, while the training time in the remaining binary experiments is 35 min. Most of the learning time is spent on training the Xception model, while a little time is spent on the residual blocks of the TCN. TCN depends mainly on the residual blocks, and the core of these blocks is the dilated convolutional layer [69]. The complexity of the dilated convolutional layer is O (n). We have employed fivefold cross-validation, whereby the training time will be multiplied by 5. The time required to extract features from the ECGConvnet model is 3.9, 1.85, and 1.55 min for the multi-class experiments, and 1.2 min for the binary-class experiments. The time taken for the classification of the features from the proposed model on all experiments is nearly the same. It is essential to know that TCN can be highly paralleled, and this gives an advantage to this model, which can be accelerated by technologies such as parallel computing.

In terms of other related works, few studies targeted the diagnosis of ECG diseases, especially COVID, using ECG images reports. Table 7 shows the papers that worked on ECG images reports for diagnosis of different diseases, especially COVID, on the two recently published datasets. Khan et al. [38] proposed a diagnosis methodology based on 12-lead-based ECG image reports. The dataset in Khan and Hussain, [36] was used in their work. The methodology was based on single shoot detection (SSD) combined with Mobile Net v2. The data consisted of four main classes: MI, PMI, normal, and abnormal. The data were divided into 80% training and 20% for the test. The total accuracy reached by this methodology was 98.33%. Ozdemir et al. (2020) proposed a new methodology based on hexaxial feature mapping by applying gray-level co-occurrence matrix (GLCM) to extract features. Then, these features were fed to a convolutional neural network (CNN) for diagnosis. The dataset in Khan and Hussain [35] was used in their work. Two main experiments were applied for the diagnosis based on fivefold cross-validation. The first experiment is the diagnosis of COVID patients and no finding individual (normal). The method achieved an accuracy of 96.20% and an F1-measure of 96.30%. The second experiment is based on the diagnosis of COVID patients as positive class and another class label as negative including normal, abnormal, and MI. The employed methodology achieved an accuracy of 93.00% and an F1-measure of 93.20%. The former two experiments are binary classification problems.

**Table 7:** Related work using ECG image reports based on deep learning methodologies

Authors	No. of classes and leads	Experiments	Methodology	Accuracy
Khan et al. (2021b)	4 classes	**Multi-class: 928 image reports SD**	Data resizing and labeling + SSD + Mobile v2	**Multi-class**: Four classes diagnosis
	12- ECG leads	MI (172) vs PMI (233) vs		80% Training 20% Test
		Normal (284) vs abnormal (233)		A = 98.33%
Ozdemir et al. (2021)	2 classes	**Binary-class**: **500 Images FD**	Hexaxial feature mapping by GLCM + CNN	Fivefold Cross-validation
	12- ECG leads	COVID (250) vs no findings		**Binary-class**: COVID vs No Findings:
		(250 Normal)		A = 96.20%, F1-measure = 96.30%
		**Binary-class: 500 images FD**		**Binary-class:** Positive vs Negative:
		For positive (250 COVID) vs negative (84 MI, 83 normal, 83 PMI, 83 abnormal)		A = 93.00%, F1-measure = 93.20%
Rahman et al. (2022)	2, 3, and 5 classes	**Binary-class: 1109 images FD**	Resnet-18	Fivefold Cross-validation
	12-ECG Leads	Normal (859) vs COVID (250)	Resnet-50	**Binary-class** diagnosis using Densenet
		**Multi-class: 1937 images FD**	Resnet101	A = 99.1%
		Normal (859) vs COVID (250) vs abnormalities (828)	InceptionV3	**Multi-class:** Three classes diagnosis using
		**Multi-class: 1937 images FD**	Densenet	Densenet A = 97.36%
		COVID (250) VS MI (77) VS PMI (203) VS normal (859) VS abnormal (548)	MobileNetV2	**Multi-class:** Five classes diagnosis using
				InceptionV3 A = 97.83%
Anwar et al. (2021)	5 classes	**Multi-class: 1937 images FD**	Efficient B3	**Multi-class:** Fivefold Cross-validation
	12- ECG Leads	COVID (250) vs MI (77) vs PMI (203) vs normal (859) vs abnormal (548)		Without augmentation:
				A = 81.8% F1-Score: 77.6%
				With augmentation:
				A: 76.4% F1-Score: 76.8%
Attallah (2022)	2 and 3 classes	**Binary-class: 500 images FD**	ECG-BiCoNet	Tenfold Cross-validation
	12-ECG Leads	Normal (250) vs COVID (250)	+	**Binary-class:** Two classes diagnosis using ECG-BiCoNet + SVM A: 98.6%
		**Multi-class: 750 images FD**	LDA, RF, SVM	**Multi-class:** Three classes diagnosis using ECG-BiCoNet + RF A: 91.2%
		Normal (250) vs COVID (250) vs abnormal (250)		
**The proposed method**	**5, 4, 3, and 2 classes**	**Multi-class: 3915 ECG images FD**	**Vgg16, Vgg19, Resnet101, Xception**	Fivefold cross-validation on each experiment
	**12 – ECG leads**	COVID (750) vs MI (747) vs PMI (812) vs normal (856) vs abnormal (750)	**ECGConvnet + Softmax, RF, MLP, SVM**	**ECGConvnet** + **SVM** showed the highest performance
		**Multi-class: 928 image reports SD**		**Multi-class Experiments**
		MI (172) vs PMI (233) vs		Five classes diagnosis: A = 99.74%
		Normal (284) vs abnormal (233)		Four classes diagnosis: A = 98.6%,
		**Multi-class: 750 images FD**		Three classes diagnosis: A = 99.1%,
		Normal (250) vs COVID (250) vs abnormalities (250)		**Binary-class Experiments**
		**4 binary-class experiments**		COVID vs (No Findings FD): A = 100%
		COVID (250) vs no findings (250) **FD**		Positive vs (Negative FD): A = 99.8%
		Positive (250) vs negative (250) **FD**		COVID vs (No Findings SD): A = 100%
		COVID (250) vs no findings (250) **SD**		Positive vs (Negative SD): A = 100%
		Positive (250) vs negative (250) **SD**		

FD: First dataset SD: Second dataset

Rahman et al. [54] presented various approaches for the diagnosis of ECG image reports from the dataset in Khan and Hussain [35]. Numerous pre-trained models were applied, such as Resnet18, Resnet50, Resnet101, InceptionV3, DenseNet201, and MobileNetv2. Several experiments were performed to determine the efficiency of the deep learning models. The first experiment is a two-class classification between normal and COVID, the second is a three-class classification between normal, COVID, and other abnormalities, and the third is a five-class classification between COVID, MI, PMI, normal, and abnormal. Densenet showed the highest accuracy performance on the first two experiments with accuracies of 99.1% and 97.36%, while InceptionV3 showed the highest performance on the third experiment with an accuracy of 97.83%. Moreover, a study was presented by Anwar et al. [4] for the diagnosis of the ECG image reports. The dataset in Khan and Hussain [35] was used in their study. The study diagnosed five types of classes: COVID, MI, PMI, normal, and abnormal. The methodology was based on the EfficientB3 deep learning model. Several augmentation methods were applied, such as flipping, cropping, scale perspective distorting, contrast, and gamma correction. The study showed that performance without augmentation is higher than with augmentation. Without augmentation, the results in terms of accuracy and F1-score achieved 81.8% and 77.6%, respectively, and decreased to 76.4% and 76.8%, respectively, with augmentation. The former two studies performed fivefold cross-validation on the data. It is also important to note that the data of the experiments in the former two studies were not balanced in the number of images in each class.

Attallah [5] proposed a new methodology for the diagnosis of COVID based on ECG image reports. The dataset in Khan and Hussain [35] was used in his study. The methodology was defined by ECG-BiCoNet,a pipeline of several deep learning models, such as Resnet, Inception, Inceptionresnet, Xception, and Densenet. The fully connected features and pooling features were extracted from the former models. The pooling features were passed to DWT, and then, the features from the fully connected and the output of the DWT were integrated. Several classifiers were applied in this study, such as linear decrement analysis (LDA), RF, and SVM. Two main experiments were performed in this study, and the data were divided into folds based on tenfold cross-validation. The first experiment was a binary-class between COVID and normal, and the second experiment was a multi-class between normal, COVID, and other abnormalities. The results showed that ECG-BiCoNet combined with SVM had the highest performance on the binary-class task with an accuracy of 98.6%, while ECG-BiCoNet combined with RF had the highest performance on the multi-class task with an accuracy of 91.2%. It is salient to show that ECG-BiCoNet is high in complexity due to the pipeline performed on the pre-trained models to obtain robust features.

Our proposed methodology applied five deep learning models with four different classifiers to evaluate the performance of the models. Seven experiments are developed based on the ECG paper-based reports. These include three multi-class diagnosis tasks, the first task diagnoses (COVID, MI, PMI, normal, and abnormal) from the first dataset, the second task diagnoses (MI, PMI, normal, and abnormal) from the second dataset, and the third task diagnoses (COVID, normal, and other abnormalities). The final experiments are binary-class tasks that diagnose (COVID vs No Findings from the first dataset), (Positive (COVID) vs Negative from the first dataset), (Positive (COVID) vs Negative from the second dataset) and COVID vs No Findings from the second dataset).

Several deep learning models and classifiers are used in the study, and all experiments are based on fivefold cross-validation. The results show that the ECGConvnet combined with SVM achieved the highest accuracy. The accuracy results of the seven experiments using ECGConvnet with SVM are 99.7%, 98.6%, 99.1%, 100%, 99.8%, 100%, and 100%, respectively. We used the same datasets applied in the former studies. The same experiment in Khan et al. [38] was conducted in our study using ECGConvnet and achieved a diagnostic accuracy of 98.60% with fivefold cross-validation, while the authors in Khan et al. [38] achieved an accuracy of 98.33% with 80% training and 20% testing. Moreover, the two experiments applied in Ozdemir et al. [50] were conducted using the proposed ECGConvnet model. The results showed an accuracy of 100% and 99.8% on the two experiments, respectively, whereas the authors in Ozdemir et al. [50] achieved an accuracy of 96.2% and 93.0%, respectively. The experiments performed by authors in Rahman et al. [54] and Anwar et al. [4] were conducted with balanced data using the same dataset in our study. The results obtained by the ECGConvnet using SVM on the former experiments showed higher performance than their results. Additionally, our ECGConvnet model achieved higher accuracy than the two experiments performed in [5] achieving accuracies of 100% and 99.05%, respectively, while the authors in Attallah [5] achieved accuracies of 98.6% and 91.2%, respectively. This shows the robustness of the proposed ECGConvnet model on different binary- and multi-class scenarios.

Advantages of the ECGConvnet model:

1. Obtaining robust features that can be classified easily by other classifiers.
2. Capability of the proposed model to differentiate between COVID-19 patients, even if it is influenced by cardiovascular changes.
3. Achieving a high classification performance in the diagnosis of different ECG diseases in comparison with other pre-trained models and other works developed in relation to ECG paper-based reports.
4. Automatic diagnosis of COVID-19 and other ECG arrhythmia based on ECG paper-based reports.

The limitation of the proposed model is its requirement of a large number of ECG paper-based reports so that the training set can be increased with images. This was specifically observed in the three multi-class experiments performed in this study. The first multi-class experiment that diagnosed five classes showed a high accuracy of 99.7% on a large number of ECG paper-based reports, while the second and the third experiments diagnosing only four and three classes, respectively, decreased the diagnosis accuracy to 98.6% and 99.05% on 928 and 750 ECG paper-based image reports, respectively. This degradation in the accuracy is due to the lack of images in the training set of each class passed to the ECGConvnet model.

## Applications

ECGConvnet can be used in various applications related to remote sensing [45], neural cognition [79], and brain-like computing. Several models are developed for these types of applications, such as BiCoss [82] and CerebelluMorphic [81], which achieve an accurate performance. It is also expected that ECGConvnet can replicate various learning models, such as action selection [78], context-dependent learning, motor learning [84], and movement disorders.

## Conclusions and Future Works

This paper presents an effective approach proposed for an automatic diagnosis of the COVID-19 patients and various types of ECG diseases. The approach is based on paper-based ECG image reports, and each ECG image report holds 12-leads. The approach depends on five DL models relying on Vgg19, Vgg16, Resnet101, Xception, and ECGConvnet. Furthermore, fivefold cross-validation is performed on 20% per fold to cover the entire data after the five folds. To evaluate the performance, a validation set was introduced to get the optimal parameters showing the highest accuracy on the validation set. Moreover, four classifiers were applied to determine the highest performance classifier on the models. Several experiments were conducted to show the robustness of the proposed approach. The proposed ECGConvnet showed the highest performance over other models on the test set. This proves the capability of the ECG to differentiate between COVID-19 patients, even if it is influenced by cardiovascular changes. This shows that the proposed diagnosis system is faster, accessible, more sensitive, and harmless. It is also more cost-effective than any other method. A set of recommendations can be adjusted for future works. Researchers can integrate the digital ECG recordings with their ECG image reports to enhance performance accuracy. Another recommendation is the ability to make the diagnosis system cloud-based or mobile-based to support decision making. Therefore, it will assist healthcare professionals and experts in the identification of diseases. It is also recommended for future work to apply different models for training, such as spike neural network (SNN). In SNN, the information is defined in the brain in the form of action potentials. SNN does not transmit information at each propagation cycle, but they transmit this information only when a membrane reaches a specific value, known as the threshold value. SNN takes space and time into consideration [80]. SNN connects neurons to the neurons nearby so that they can perform a separate process on the input blocks, and this is similar to the convolution and dilated convolutional layers using filters in the ECGConvnet. SNN also encodes information in the form of a pulse train so that the information cannot be lost during the binary encoding. The former process evades the large complexity caused by recurrent neural networks [83]. Even though SNN models are still behind ANNs in terms of accuracy, the gap between them is decreasing and perhaps nonexistent in some tasks.

## Data Availability

The dataset analyzed during the current study is available in the repository [ECG Images dataset of Cardiac and COVID-19 Patients’ dataset https://data.mendeley.com/datasets/gwbz3fsgp8/1] [ECG Images dataset of Cardiac Patients https://data.mendeley.com/datasets/gwbz3fsgp8/2].
